# Supplementary material for: Th2 cytokine bias induced by silver nanoparticles in peripheral blood mononuclear cells of common bottlenose dolphins (Tursiops truncatus)
Source: PeerJ. 2018 Sep 17;6:e5432. doi: 10.7717/peerj.5432 (PMC6147119; doi:10.7717/peerj.5432)

Size Distribution Report by Intensity

v2.2

Malvern

Sample Details

Sample Name: AgNPs20-3 1

SOP Name: mansettings.nano

General Notes:

**File Name:**	20170823.dts	**Dispersant Name:**	citrate buffer
**Record Number:**	7	**Dispersant RI:**	1.330
**Material RI:**	1.59	**Viscosity (cP):**	0.8878
**Material Absorbtion:**	0.010		

System

**Temperature (°C):**	25.0	**Duration Used (s):**	60
**Count Rate (kcps):**	273.9	**Measurement Position (mm):**	4.65
**Cell Description:**	Disposable sizing cuvette	**Attenuator:**	6

Results

			Size (d.n...	% Intensity:	St Dev (d.n...
**Z-Average (d.nm):**	26.40	**Peak 1:**	30.08	100.0	10.53
**PdI:**	0.125	**Peak 2:**	0.000	0.0	0.000
**Intercept:**	0.906	**Peak 3:**	0.000	0.0	0.000

Result quality Good

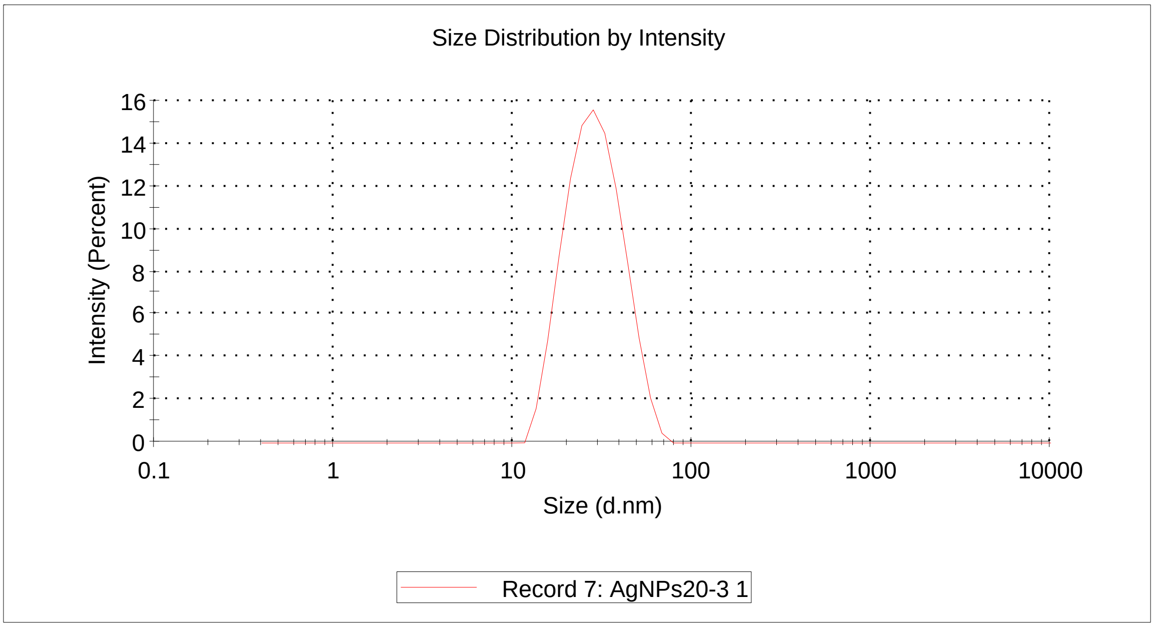

Size Distribution Report by Intensity

v2.2

Malvern

Sample Details

Sample Name: AgNPs20-3 2

SOP Name: mansettings.nano

General Notes:

**File Name:**	20170823.dts	**Dispersant Name:**	citrate buffer
**Record Number:**	8	**Dispersant RI:**	1.330
**Material RI:**	1.59	**Viscosity (cP):**	0.8878
**Material Absorbtion:**	0.010		

System

**Temperature (°C):**	25.0	**Duration Used (s):**	60
**Count Rate (kcps):**	274.9	**Measurement Position (mm):**	4.65
**Cell Description:**	Disposable sizing cuvette	**Attenuator:**	6

Results

			Size (d.n...	% Intensity:	St Dev (d.n...
**Z-Average (d.nm):**	26.71	**Peak 1:**	30.23	100.0	10.28
**PdI:**	0.120	**Peak 2:**	0.000	0.0	0.000
**Intercept:**	0.905	**Peak 3:**	0.000	0.0	0.000

Result quality Good

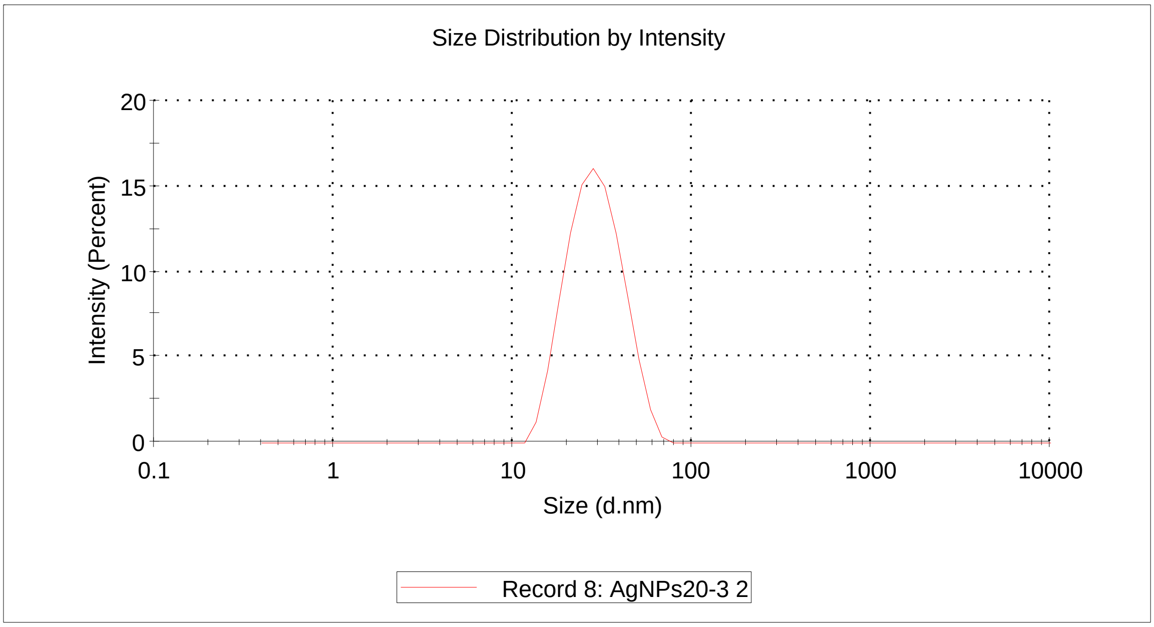

Size Distribution Report by Intensity

v2.2

Sample Details

Sample Name: AgNPs20-3 3

SOP Name: mansettings.nano

General Notes:

**File Name:**	20170823.dts	**Dispersant Name:**	citrate buffer
**Record Number:**	9	**Dispersant RI:**	1.330
**Material RI:**	1.59	**Viscosity (cP):**	0.8878
**Material Absorbtion:**	0.010		

System

**Temperature (°C):**	25.0	**Duration Used (s):**	60
**Count Rate (kcps):**	274.6	**Measurement Position (mm):**	4.65
**Cell Description:**	Disposable sizing cuvette	**Attenuator:**	6

Results

			Size (d.n...	% Intensity:	St Dev (d.n...
**Z-Average (d.nm):** 26.74		**Peak 1:**	30.51	100.0	10.98
**PdI:** 0.126		**Peak 2:**	0.000	0.0	0.000
**Intercept:** 0.904		**Peak 3:**	0.000	0.0	0.000

Result quality Good

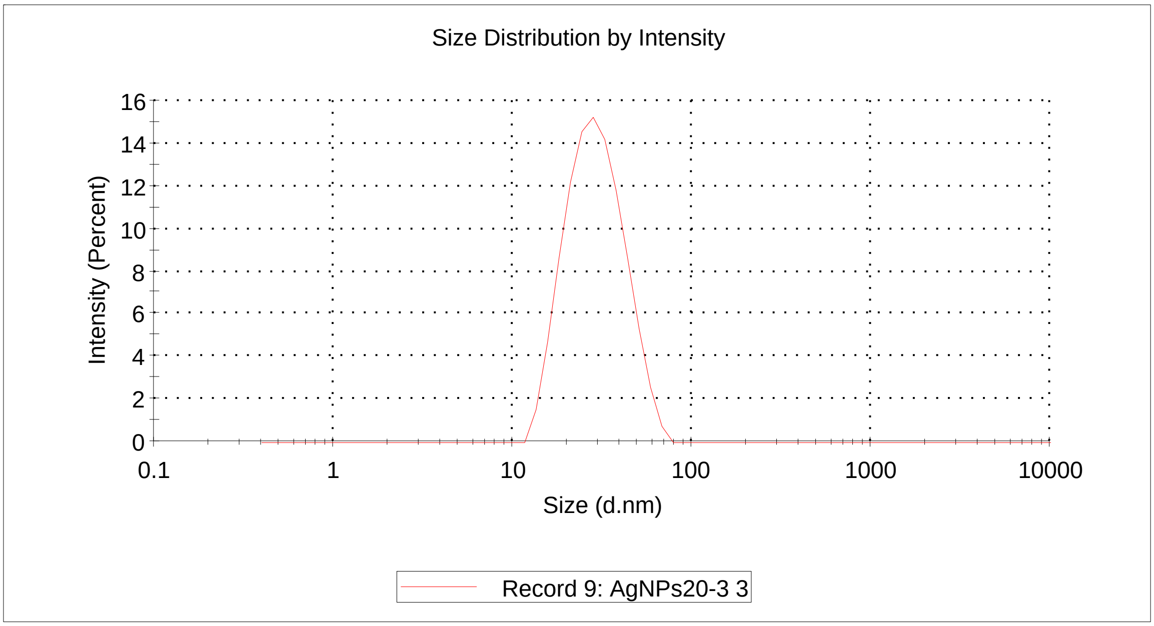

Zeta Potential Report
v2.3

Malvern Instruments Ltd - © Copyright 2008

Sample Details

Sample Name: AgNPs20-5 1

SOP Name: mansettings.nano

General Notes:

File Name: 20170823.dts

Record Number: 13

Dispersant Name: citrate buffer

Dispersant RI: 1.330

Viscosity (cP): 0.8878

Dispersant Dielectric Constant: 78.5

System

Temperature (°C): 25.0

Count Rate (kcps): 144.3

Cell Description: Clear disposable zeta cell

Zeta Runs: 13

Measurement Position (mm): 2.00

Attenuator: 9

Results

			Mean (mV)	Area (%)	St Dev (mV)
**Zeta Potential (mV):**	-40.1	**Peak 1:**	-30.8	62.9	11.0
**Zeta Deviation (mV):**	26.4	**Peak 2:**	-56.2	14.4	5.35
**Conductivity (mS/cm):**	0.686	**Peak 3:**	-73.5	9.6	6.28
**Result quality**	**See result quality report**				

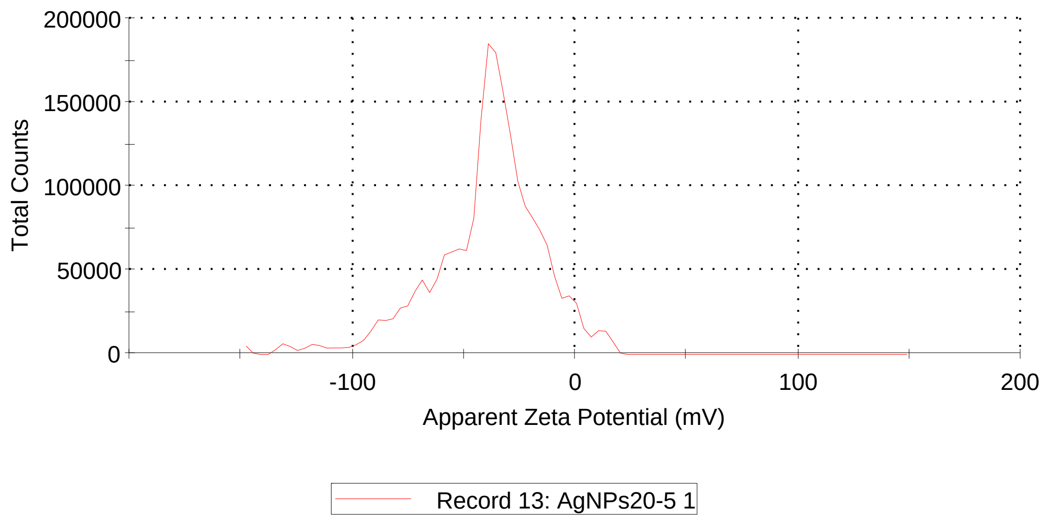

Zeta Potential Report

v2.3

Malvern Instruments Ltd - © Copyright 2008

Sample Details

Sample Name: AgNPs20-5 2

SOP Name: mansettings.nano

General Notes:

File Name: 20170823.dts

Record Number: 14

Dispersant Name: citrate buffer

Dispersant RI: 1.330

Viscosity (cP): 0.8878

Dispersant Dielectric Constant: 78.5

System

Temperature (°C): 25.0

Count Rate (kcps): 311.3

Cell Description: Clear disposable zeta cell

Zeta Runs: 12

Measurement Position (mm): 2.00

Attenuator: 9

Results

			Mean (mV)	Area (%)	St Dev (mV)
**Zeta Potential (mV):**	-37.1	**Peak 1:**	-49.8	41.3	10.8
**Zeta Deviation (mV):**	19.9	**Peak 2:**	-30.4	36.9	4.95
**Conductivity (mS/cm):**	0.709	**Peak 3:**	-12.6	19.3	6.81

Result quality See result quality report

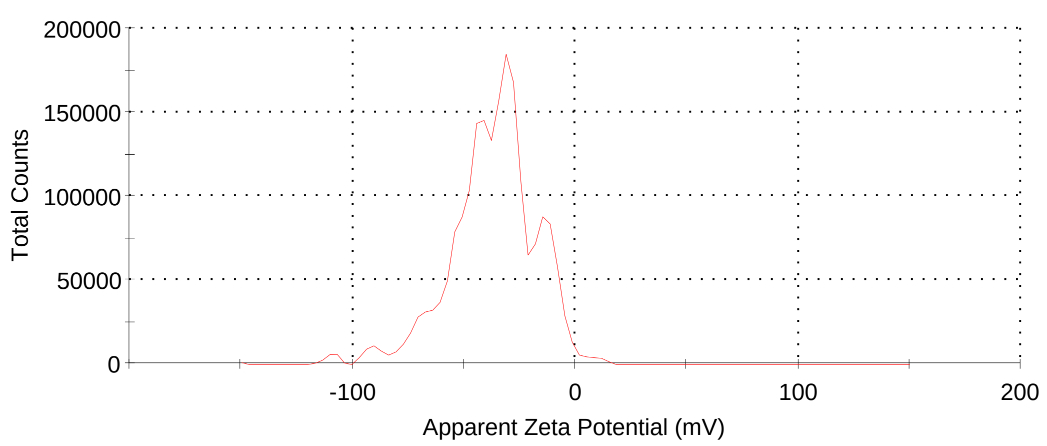

Zeta Potential Report
v2.3

Malvern Instruments Ltd - © Copyright 2008

Sample Details

Sample Name: AgNPs20-5 3

SOP Name: mansettings.nano

General Notes:

File Name: 20170823.dts

Record Number: 15

Dispersant Name: citrate buffer

Dispersant RI: 1.330

Viscosity (cP): 0.8878

Dispersant Dielectric Constant: 78.5

System

Temperature (°C): 25.0

Count Rate (kcps): 131.0

Cell Description: Clear disposable zeta cell

Zeta Runs: 12

Measurement Position (mm): 2.00

Attenuator: 9

Results

			Mean (mV)	Area (%)	St Dev (mV)
**Zeta Potential (mV):**	-39.7	**Peak 1:**	-37.8	59.4	9.17
**Zeta Deviation (mV):**	23.1	**Peak 2:**	-14.5	20.7	5.22
**Conductivity (mS/cm):**	0.722	**Peak 3:**	-74.6	8.2	6.24

Result quality See result quality report

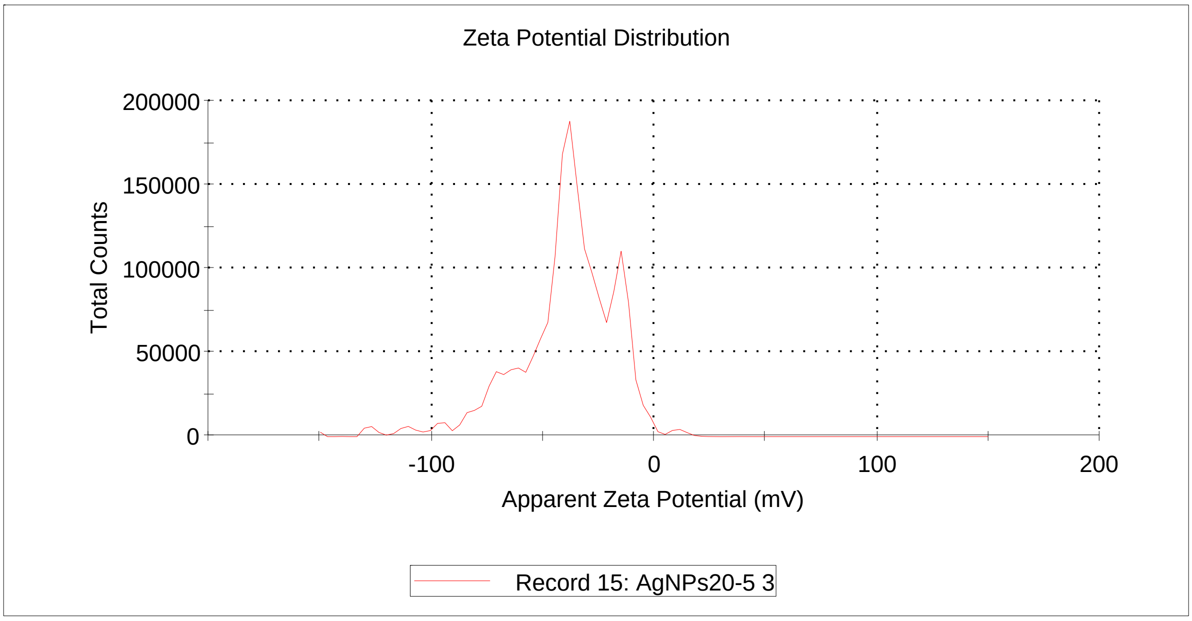

Size Distribution Report by Intensity

v2.2

Malvern

Sample Details

Sample Name: AgNPs20-1 1

SOP Name: mansettings.nano

General Notes:

**File Name:**	20170823.dts	**Dispersant Name:**	citrate buffer
**Record Number:**	1	**Dispersant RI:**	1.330
**Material RI:**	1.59	**Viscosity (cP):**	0.8878
**Material Absorbtion:**	0.010		

System

**Temperature (°C):**	25.0	**Duration Used (s):**	60
**Count Rate (kcps):**	284.8	**Measurement Position (mm):**	1.25
**Cell Description:**	Disposable sizing cuvette	**Attenuator:**	5

Results

			Size (d.n...	% Intensity:	St Dev (d.n...
**Z-Average (d.nm):**	26.59	**Peak 1:**	29.56	100.0	9.212
**PdI:**	0.105	**Peak 2:**	0.000	0.0	0.000
**Intercept:**	0.910	**Peak 3:**	0.000	0.0	0.000

Result quality Good

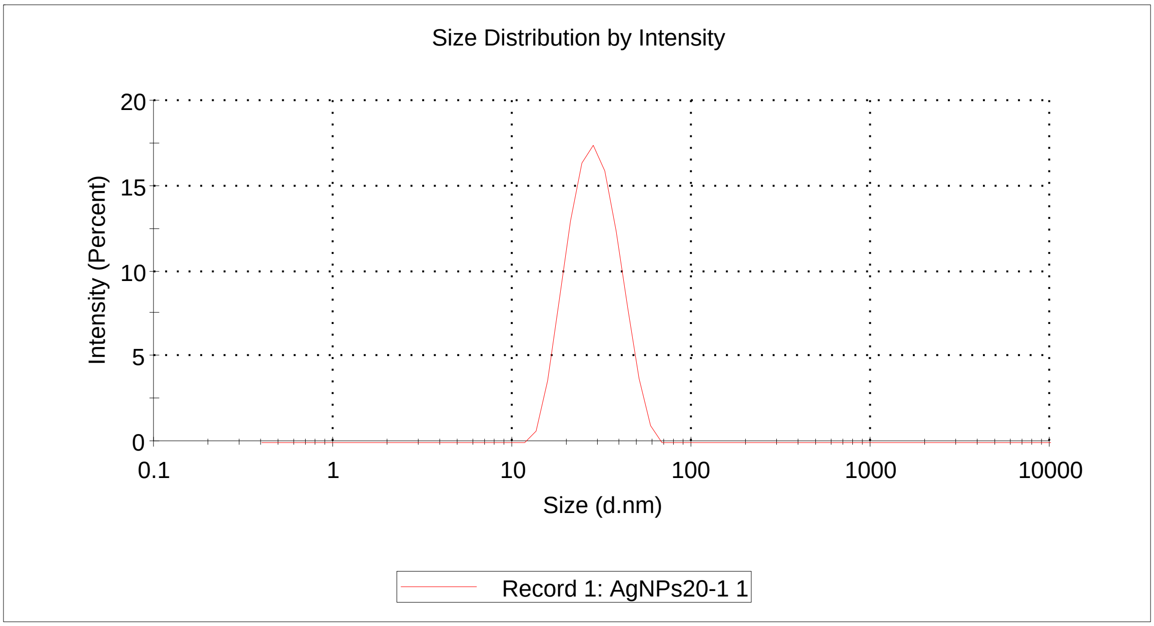

Size Distribution Report by Intensity

v2.2

Malvern

Sample Details

**Sample Name:**	AgNPs20-1 2
**SOP Name:**	mansettings.nano
**General Notes:**	

**File Name:**	20170823.dts	**Dispersant Name:**	citrate buffer
**Record Number:**	2	**Dispersant RI:**	1.330
**Material RI:**	1.59	**Viscosity (cP):**	0.8878
**Material Absorbtion:**	0.010		

System

**Temperature (°C):**	25.0	**Duration Used (s):**	60
**Count Rate (kcps):**	284.0	**Measurement Position (mm):**	1.25
**Cell Description:**	Disposable sizing cuvette	**Attenuator:**	5

Results

			**Size (d.n...**	**% Intensity:**	**St Dev (d.n...**
**Z-Average (d.nm):**	26.43	**Peak 1:**	29.32	100.0	9.045
**PdI:**	0.100	**Peak 2:**	0.000	0.0	0.000
**Intercept:**	0.909	**Peak 3:**	0.000	0.0	0.000
**Result quality**	**Good**				

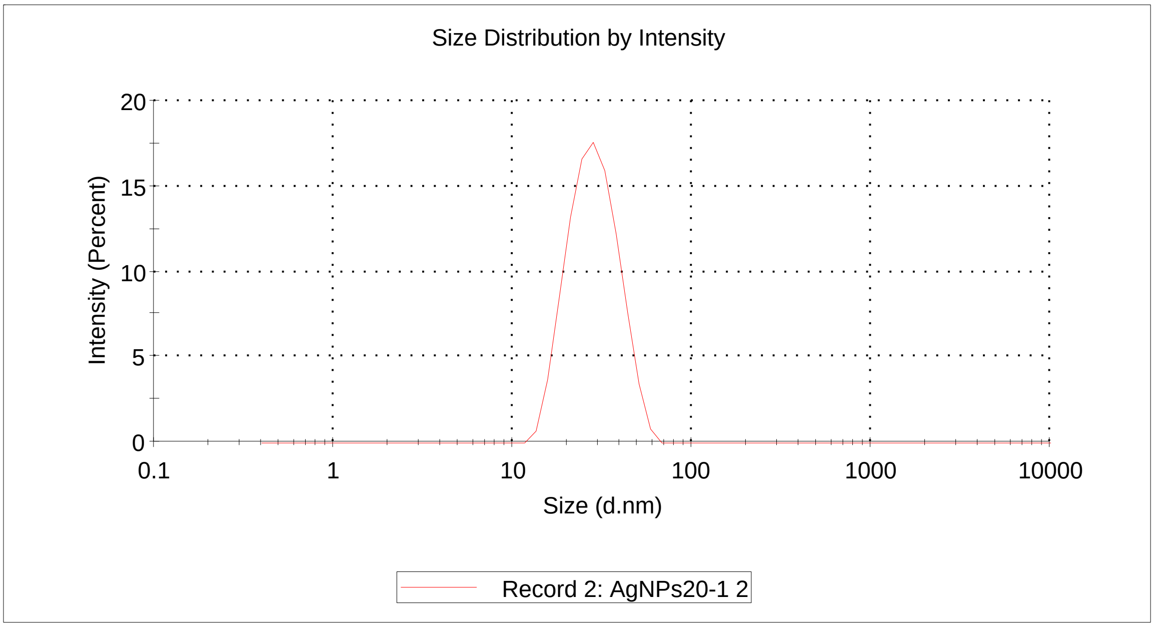

Size Distribution Report by Intensity

v2.2

Sample Details

Sample Name: AgNPs20-1 3

SOP Name: mansettings.nano

General Notes:

**File Name:**	20170823.dts	**Dispersant Name:**	citrate buffer
**Record Number:**	3	**Dispersant RI:**	1.330
**Material RI:**	1.59	**Viscosity (cP):**	0.8878
**Material Absorbtion:**	0.010		

System

**Temperature (°C):**	25.0	**Duration Used (s):**	60
**Count Rate (kcps):**	288.4	**Measurement Position (mm):**	1.25
**Cell Description:**	Disposable sizing cuvette	**Attenuator:**	5

Results

			Size (d.n...	% Intensity:	St Dev (d.n...
**Z-Average (d.nm):**	26.59	**Peak 1:**	30.05	100.0	10.19
**PdI:**	0.122	**Peak 2:**	0.000	0.0	0.000
**Intercept:**	0.911	**Peak 3:**	0.000	0.0	0.000

Result quality **Good**

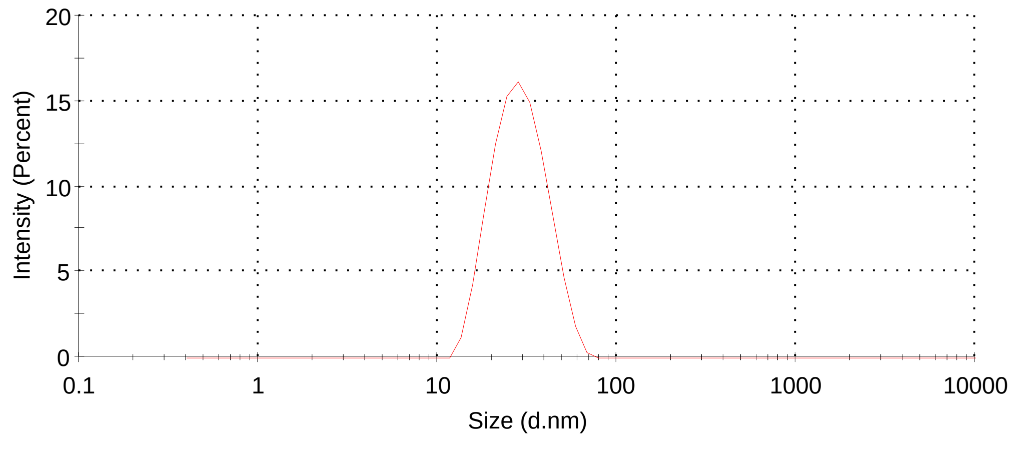

Zeta Potential Report

v2.3

Malvern Instruments Ltd - © Copyright 2008

Sample Details

Sample Name: AgNPs20-4 1

SOP Name: mansettings.nano

General Notes:

File Name: 20170823.dts

Record Number: 10

Dispersant Name: citrate buffer

Dispersant RI: 1.330

Viscosity (cP): 0.8878

Dispersant Dielectric Constant: 78.5

System

Temperature (°C): 25.0

Count Rate (kcps): 116.1

Cell Description: Clear disposable zeta cell

Zeta Runs: 15

Measurement Position (mm): 2.00

Attenuator: 7

Results

			Mean (mV)	Area (%)	St Dev (mV)
**Zeta Potential (mV):**	-43.1	**Peak 1:**	-39.5	88.6	12.4
**Zeta Deviation (mV):**	16.6	**Peak 2:**	-72.3	9.8	7.23
**Conductivity (mS/cm):**	0.690	**Peak 3:**	-97.8	1.5	5.15

Result quality **See result quality report**

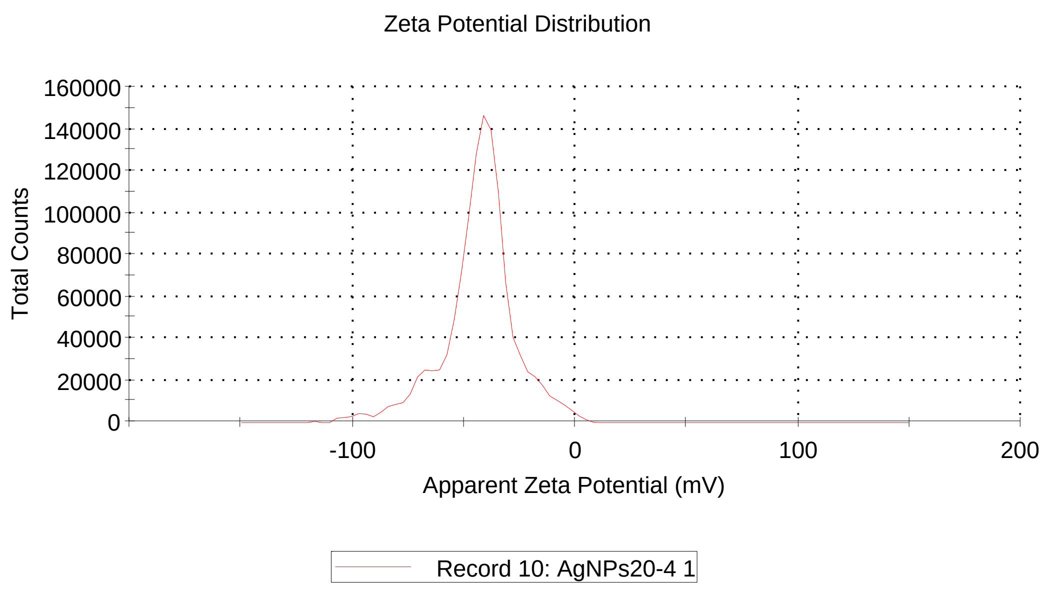

Zeta Potential Report

v2.3

Malvern Instruments Ltd - © Copyright 2008

Sample Details

Sample Name: AgNPs20-4 2

SOP Name: mansettings.nano

General Notes:

File Name: 20170823.dts

Record Number: 11

Dispersant Name: citrate buffer

Dispersant RI: 1.330

Viscosity (cP): 0.8878

Dispersant Dielectric Constant: 78.5

System

Temperature (°C): 25.0

Count Rate (kcps): 41.1

Cell Description: Clear disposable zeta cell

Zeta Runs: 12

Measurement Position (mm): 2.00

Attenuator: 7

Results

			Mean (mV)	Area (%)	St Dev (mV)
**Zeta Potential (mV):**	-43.4	**Peak 1:**	-37.2	69.8	10.6
**Zeta Deviation (mV):**	21.5	**Peak 2:**	-62.0	12.2	4.35
**Conductivity (mS/cm):**	0.714	**Peak 3:**	-81.7	12.0	9.65

Result quality **See result quality report**

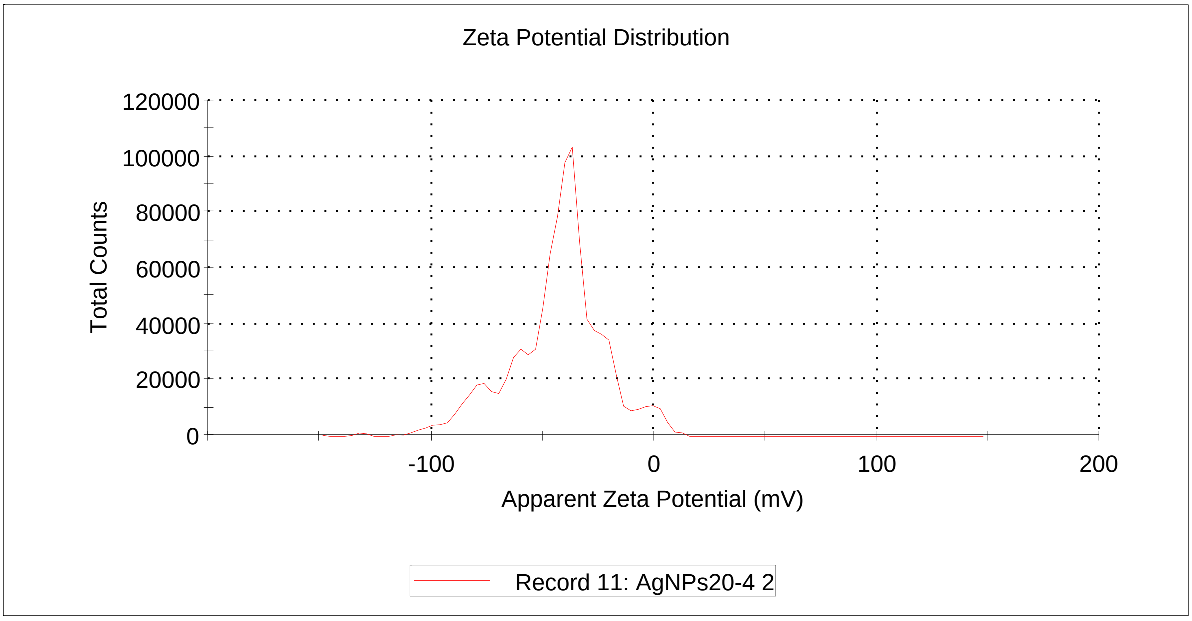

Zeta Potential Report

v2.3

Malvern Instruments Ltd - © Copyright 2008

Sample Details

Sample Name: AgNPs20-4 3

SOP Name: mansettings.nano

General Notes:

File Name: 20170823.dts

Record Number: 12

Dispersant Name: citrate buffer

Dispersant RI: 1.330

Viscosity (cP): 0.8878

Dispersant Dielectric Constant: 78.5

System

Temperature (°C): 25.0

Count Rate (kcps): 69.1

Cell Description: Clear disposable zeta cell

Zeta Runs: 12

Measurement Position (mm): 2.00

Attenuator: 7

Results

			Mean (mV)	Area (%)	St Dev (mV)
**Zeta Potential (mV):**	-46.1	**Peak 1:**	-32.7	34.5	7.11
**Zeta Deviation (mV):**	21.4	**Peak 2:**	-45.6	33.2	4.32
**Conductivity (mS/cm):**	0.725	**Peak 3:**	-61.2	19.9	6.73

Result quality See result quality report

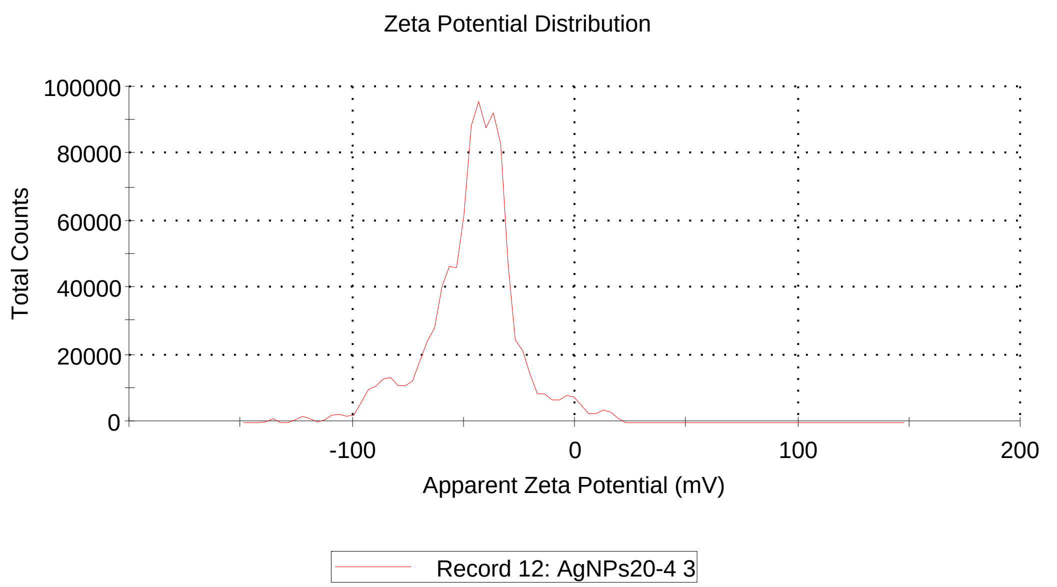

Supplement: Supplemental Information 1 [file peerj-06-5432-s001.pdf]
