# Supplementary figures and images for: Th2 cytokine bias induced by silver nanoparticles in peripheral blood mononuclear cells of common bottlenose dolphins (Tursiops truncatus)
Source: PeerJ. 2018 Sep 17;6:e5432. doi: 10.7717/peerj.5432 (PMC6147119; doi:10.7717/peerj.5432)

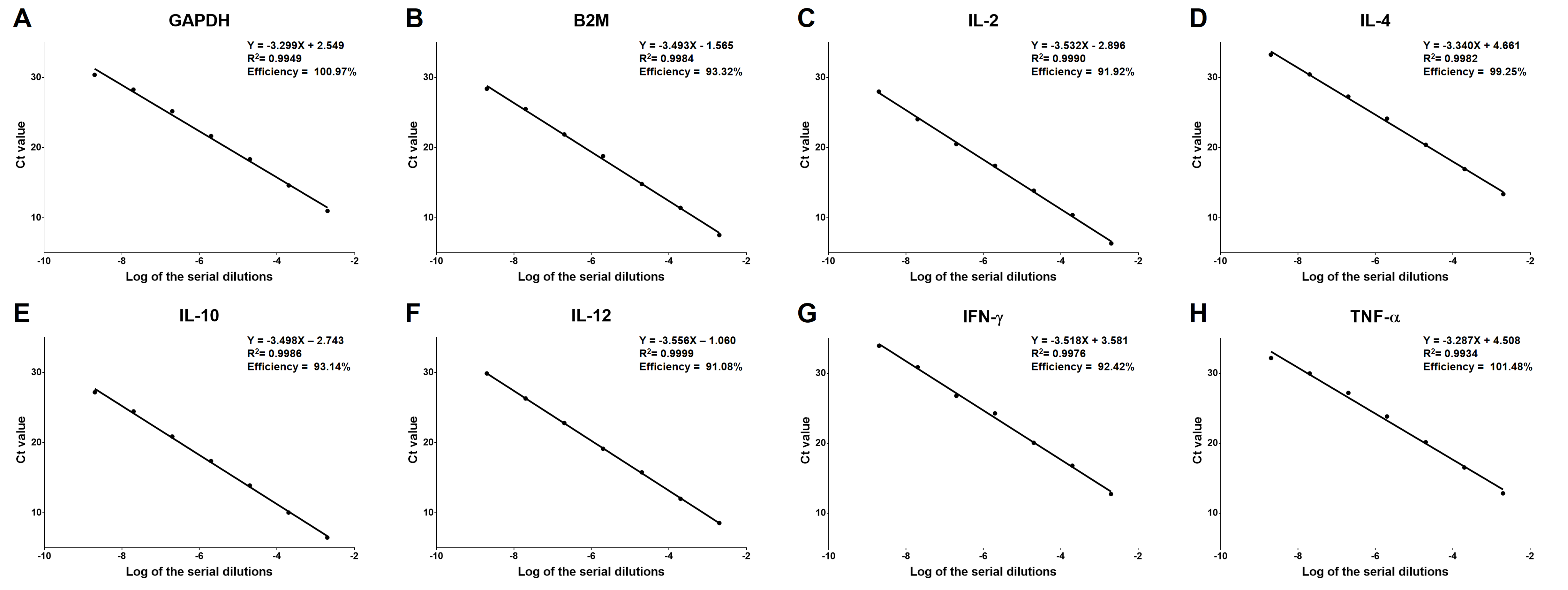

Supplement: Figure S1 [file peerj-06-5432-s004.png]
